# On the origin of F-wave: involvement of central synaptic mechanisms

**DOI:** 10.1093/brain/awad342

**Published:** 2023-10-05

**Authors:** M Görkem Özyurt, Filipe Nascimento, Robert M Brownstone, Marco Beato

**Affiliations:** Department of Neuroscience Physiology and Pharmacology (NPP), University College London, London WC1E 6BT, UK; Department of Neuromuscular Diseases, UCL Queen Square Institute of Neurology, University College London, London WC1N 3BG, UK; Department of Neuroscience Physiology and Pharmacology (NPP), University College London, London WC1E 6BT, UK; Department of Neuromuscular Diseases, UCL Queen Square Institute of Neurology, University College London, London WC1N 3BG, UK; Department of Neuromuscular Diseases, UCL Queen Square Institute of Neurology, University College London, London WC1N 3BG, UK; Department of Neuroscience Physiology and Pharmacology (NPP), University College London, London WC1E 6BT, UK

**Keywords:** motor neurons, recurrent excitation, F-wave, synapse, spinal cord

## Abstract

Neurophysiological methods are used widely to gain information about motor neuron excitability and axon conduction in neurodegenerative diseases. The F-wave is a common biomarker used to test motor neuron properties in the diagnosis of neurological diseases. Although the origin of the F-wave is a subject of debate, the most widely accepted mechanism posits that the F-wave is generated by the backfiring of motor neurons stimulated antidromically from the periphery.

In this study, we developed an *ex vivo* mouse sciatic nerve-attached spinal cord preparation with sensory axons severed.

In this preparation, stimulation of the whole sciatic nerve or its tibial branch evoked responses with the electrophysiological signatures of F-waves. Manipulations of synaptic transmission by either removal of extracellular calcium or block of post-synaptic glutamate receptors abolished these responses.

These results suggest that F-waves are mediated by spinal microcircuits activated by recurrent motor axon collaterals via glutamatergic synapses.

## Teaser

Özyurt *et al*. investigate the origin of a widely used clinical tool known as the F-wave and show that F-waves are mediated by spinal microcircuits activated by recurrent motor axon collaterals via central glutamatergic synapses. These findings could change the interpretation of pathological changes detected using F-waves.

## Introduction

Neurological disorders that affect motor systems, such as amyotrophic lateral sclerosis (ALS) and peripheral neuropathies, lead to substantial alterations in the properties of spinal motor neurons. In clinical neurophysiology, electromyographic (EMG) recordings are commonly employed to diagnose and understand the progression of such conditions. Electrophysiological parameters such as F-wave amplitude and latency, first described by Magladery and McDougal,^1^ are important measurements of motor neuron and motor nerve excitability. Typically, F-waves have low and variable amplitude (2–5% of the maximal direct motor response), variable latency and are subject to failures.^2^ F-waves are often used to estimate motor axon conduction velocity and provide valuable information about a wide range of motor disorders such as polyneuropathies and demyelinating conditions.^2,3^

The amplitude and latency of F-waves are both clinically useful properties. For example, a sensitive marker of abnormalities in lumbosacral radiculopathies^4^ is chronodispersion: the difference between minimal and maximal F-wave latencies in response to a given stimulus. And the amplitude, duration and probability of occurrence of F-waves are used to gauge the excitability state of motor pools in a variety of CNS disorders.^5-8^ For example, in the early stages of ALS, alterations in the excitability of motor neurons^9^ correlate with increased amplitude of the F-wave.^8^

Although F-waves are used routinely for diagnosing and understanding a variety of neurological disorders, the mechanisms underlying this delayed evoked response are not fully understood. The prevalent explanation for the origin of the F-wave is that the antidromic spikes elicited by electrical stimulation of motor axons lead to somatic depolarization in a variable minority of motor neurons, which in turn leads to the generation of a second spike that is propagated orthodromically and thus recorded as a low amplitude motor response (i.e. F-wave).^2,10^ While this is the generally accepted mechanism for the generation of F-waves, it is difficult to reconcile the concept of rebound spikes with the inactivation kinetics of sodium channels that presumably would prevent the generation (or propagation) of a second spike following the initial antidromic invasion. It is also notable that antidromic stimulation has been used to identify motor neurons in a variety of experimental systems *in vivo* and *in vitro,*^11,12^ but the occurrence of a second ‘rebound’ spike has never been reported.

In this report, we investigated an alternative explanation for the generation of F-waves based on anatomical studies demonstrating that motor neurons in cats are synaptically connected with other motor neurons^13,14^ and functional studies showing that motor neurons in mice are synaptically connected with other motor neurons. Thus, antidromic activation of axons leads to excitation of other motor neurons (either directly or via excitatory interneurons^12,15-17^) via recurrent motor axon collaterals, a phenomenon known as recurrent excitation.^12,15^ This excitation, mediated by glutamatergic transmission, can lead to orthodromic action potentials.^12^ We thus hypothesized that recurrent excitation would be detected as a low-amplitude motor response in muscle recordings and could thus underlie F-waves. Since experiments in humans are not amenable to pharmacological manipulations, we tested this hypothesis using *ex vivo* preparations of neonatal mouse spinal cords with sciatic nerves attached. This preparation allowed us to evoke and record the equivalent of F-waves directly from the nerves while retaining the ability to use pharmacological manipulations. We present evidence that supports the hypothesis that F-waves result from recurrent excitation of motor neurons.

## Materials and methods

All the procedures were conducted in accordance with the Animal (Scientific Procedures) Act (Home Office, UK, 1986) and were approved by the UCL Ethical Committee under project license number PP2688499. *Ex vivo* experiments with nerve attached were performed on tissue obtained from female (*n* = 7) or male (*n* = 4) mice on postnatal Days 1–3 (P1–3). Patch clamp experiments on dorsal horn ablated spinal cords were done in female (*n* = 4) and male (*n* = 6) wild-type mice bred on a C57Bl/6J background at P5–13. Full details of the methods are provided in the Supplementary material.

## Results

**Figure 1:**
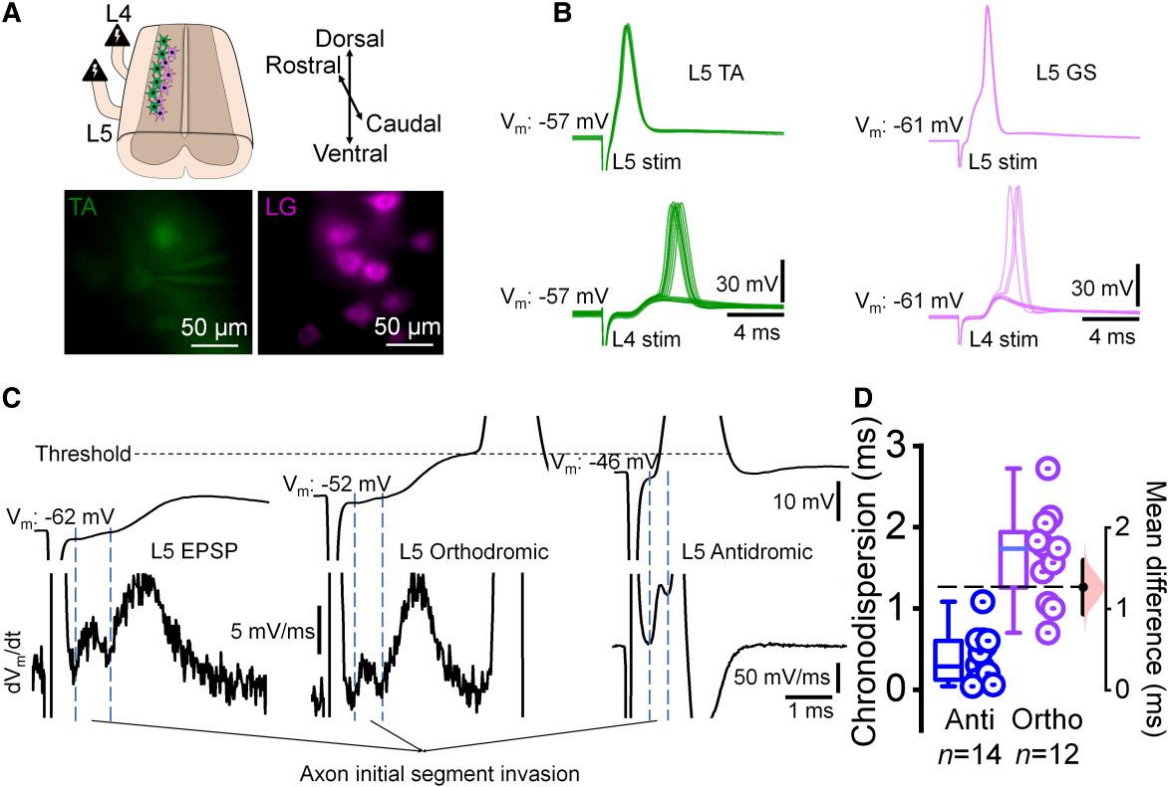
**Ortho- and antidromic action potentials generated in motor neurons following stimulation of ventral roots.** (**A**) Drawing of the dorsal horn ablated spinal cord preparation with L4 and L5 ventral root stimulation (*top*) and representative images of labelled tibialis anterior (TA) and gastrocnemius (GS) motor neurons. (**B**) Current clamp recordings of ventral root evoked responses in one TA (*left*, five sweeps) and one GS (*right*, two sweeps) motor neuron from postnatal Day 5–13 mice, showing homosegmentally-evoked antidromic spikes (*top*). At resting membrane potential, orthodromic spikes and occasional spike failures with visible excitatory post-synaptic potentials (EPSPs) were recorded in response to adjacent root stimulation (*bottom*, 14 sweeps for TA and 9 sweeps for GS). (**C**) Current clamp recordings of L5 ventral root evoked responses in unlabelled dorsolateral motor neurons (*top*) showing subthreshold EPSP, near threshold EPSP-evoked orthodromic spike and antidromic spike. *Bottom*: Derivatives showing antidromic axon initial segment spikes even in the absence of somatic antidromic spikes. (**D**) Plot of chronodispersion of antidromic (anti) and orthodromic (ortho) spikes. Each point indicates a motor neuron. *n* = number of motor neurons. *Right*: Effect size estimation plot. stim = stimulation; V_m_ = membrane potential.

We recorded motor neurons innervating either the lateral gastrocnemius (LG) or tibialis anterior (TA) muscle in dorsal horn ablated spinal cords and stimulated ventral roots in the same or adjacent segment to the recorded motor neuron (Fig. 1A). In all cases, an antidromic spike was observed following same segment stimulation (Fig. 1B, upper traces), but the subsequent excitatory post-synaptic potential (EPSP) was never sufficient to evoke an orthodromic spike following the antidromic one. On the contrary, stimulation of the adjacent segment invariably resulted in an EPSP that could exceed threshold and give rise to an orthodromic spike (Fig. 1B, lower traces).

We next attempted to prevent antidromic invasion of the soma by hyperpolarizing the motor neurons, and to determine whether this block would lead to the appearance of an orthodromic spike. Indeed, in 14 of 25 recorded motor neurons with antidromic spikes, hyperpolarization (between −60 and −75 mV) by direct current injection prevented somatodendritic (SD) antidromic spike but not the axon initial segment (IS) spike (Fig. 1C, left), visible as a peak in the first derivative of the voltage trace (Fig. 1C, left, bottom row). This IS spike was followed by an EPSP that did not reach threshold. Reducing the injected current (Fig. 1C, middle) enabled the EPSP to reach threshold and generate an orthodromic spike. But when the motor neuron was held at its resting membrane potential (Fig. 1C, right) the occurrence of the antidromic spike prevented the occurrence of the orthodromic one. We also compared the chronodispersion of the anti and orthodromic spikes recorded and found a greater degree of chronodispersion for the orthodromic spikes than for the antidromic ones (Fig. 1D; 1.66 ± 0.55 ms versus 0.39 ± 0.29 ms). This was not unexpected, given the EPSP reaches the threshold at different times during its rising phase.

**Figure 2:**
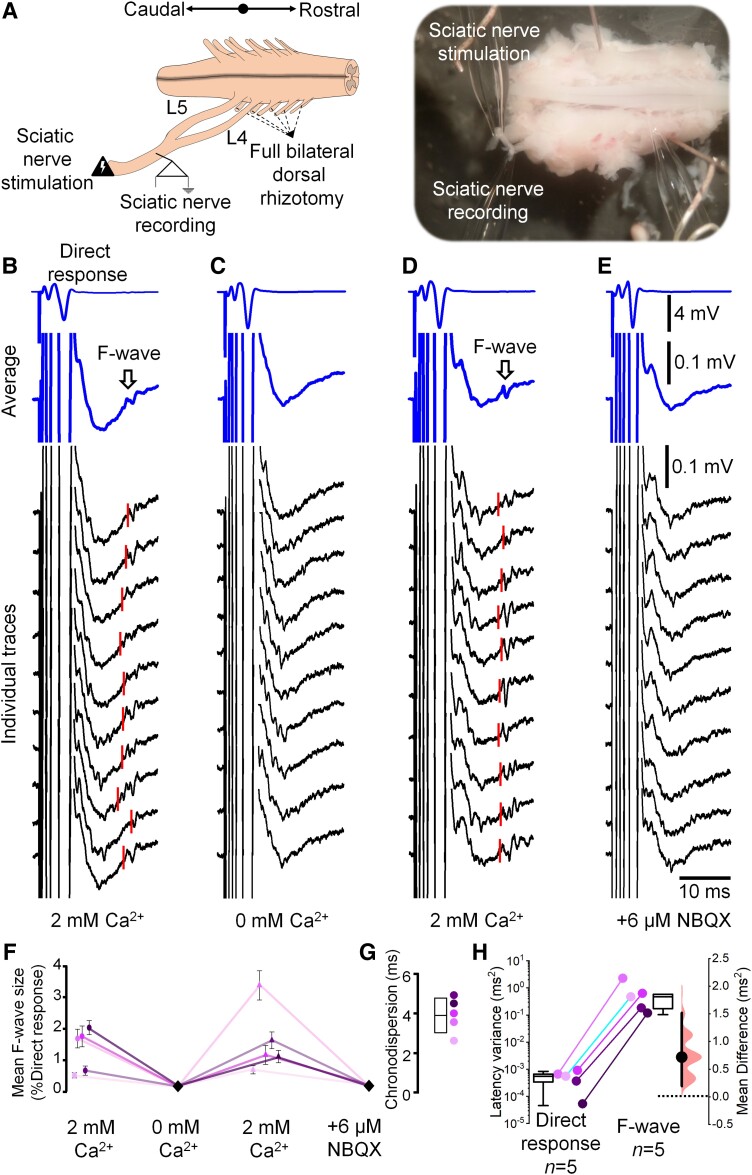
**F-waves recorded from neonatal mouse spinal cord–sciatic nerve *ex vivo* preparations following sciatic nerve stimulation depend on central synaptic release mechanisms**. (**A**) Schematic (*left*) and representative photograph (*right*) of the isolated spinal cord–sciatic nerve preparation, depicting the sites of stimulation (distal sciatic nerve) and recording (proximal sciatic nerve). (**B**) Example of averaged (blue trace, without peak alignment) and individual (black) traces obtained from a 2-day-old male mouse pup. Direct responses are amplified in the second averaged trace to visualize the long latency F-wave, indicated by arrows. Red lines show the estimated onset of F-waves. (**C**) Ca^2+^-free artificial CSF abolished the F-wave that (**D**) was restored upon reperfusion with a solution containing 2 mM Ca^2+^. Red lines show the estimated onset of F-waves. (**E**) Perfusion with the AMPA receptor antagonist NBQX also abolished the F-wave. (**F**) Plots show mean F-wave amplitudes in each animal (different colours) in different conditions and (**G**) chronodispersion of F-wave in the control 2 mM Ca^2+^ condition (mean ± standard deviation). (**H**) The latency variance of the direct responses and F-waves shows high variance in F-wave latencies (note log_10_ scale). *n* = number of *ex vivo* preparations (animals). *Right*: Effect size estimation plot.

We reasoned that in an intact system these orthodromic spikes could give rise to a delayed motor response propagating along the nerves and thus account for F-waves. We next tested if we could evoke and measure a motor response that would have the electrophysiological signatures of the F-wave (Fig. 2A). We stimulated the whole sciatic nerve (P1–3, *n* = 5) and detected a large, early direct response at a more proximal sciatic site (Fig. 2B). Following this direct response, we detected a subsequent response with a latency of 29.1 ± 5.6 ms, with 3.9 ± 0.8 ms chronodispersion (CV: 0.58 ± 0.08 m/s, in the *n* = 3 cords in which the orientation of the nerve allowed accurate length determination). Our CV estimate is similar to the reported neonatal mice motor axon CV.^18^ This second response could be regarded as an *ex vivo* analogue of the F-wave that is usually measured through EMG since it: (i) followed an initial direct motor volley; (ii) did not arise from sensory axon mediated reflexes; and (iii) was variable in size, shape and latency (see individual sweeps in Fig. 2B–E, chronodispersion in Fig. 2G and latency variance in Fig. 2H). Since these features matched the characteristics of the F-wave studied in clinical neurophysiology, we concluded that in our *ex vivo* neonatal mouse preparations, we were able to successfully evoke and measure F-waves from nerves.

Given our postulate that orthodromic action potentials lead to F-waves (Fig. 1) and given that we could record an F-wave equivalent (Fig. 2), we were now able to ask whether F-waves are generated by synaptic mechanisms. That is, could we abolish the F-wave response by manipulating synaptic transmission at either pre- or post-synaptic sites?

We first lowered synaptic release probability by removing Ca^2+^ from the extracellular solution. Doing so did not reduce the direct response (∼4% increase compared with control condition) but completely abolished the F-wave (Fig. 2C). This effect was reversible: following re-equilibration with 2 mM extracellular Ca^2+^; the F-wave recovered (Fig. 2D), indicating that blocking pre-synaptic transmitter release by removing Ca^2+^ is sufficient to prevent the generation of the F-wave.

Since Ca^2+^ removal could also modulate motor neuron excitability, we next blocked post-synaptic receptors. Since glutamate (AMPA) receptors are known to mediate recurrent excitation between motor neurons,^12^ and blocking them would not affect motor neuron excitability,^12^ we used a selective AMPA antagonist, NBQX. We found that exogenous application of NBQX (6 µM) completely suppressed F-waves without affecting the direct response (∼2% increase compared with control condition) (Fig. 2E). That is, we found that F-waves can be completely suppressed by either impairing the synaptic release machinery (reducing the probability of release by removing Ca^2+^) or by blocking post-synaptic receptors (Fig. 2F). These results can be explained if F-waves are generated by recurrent synaptic connections but not if they are generated by rebound spikes.

**Figure 3:**
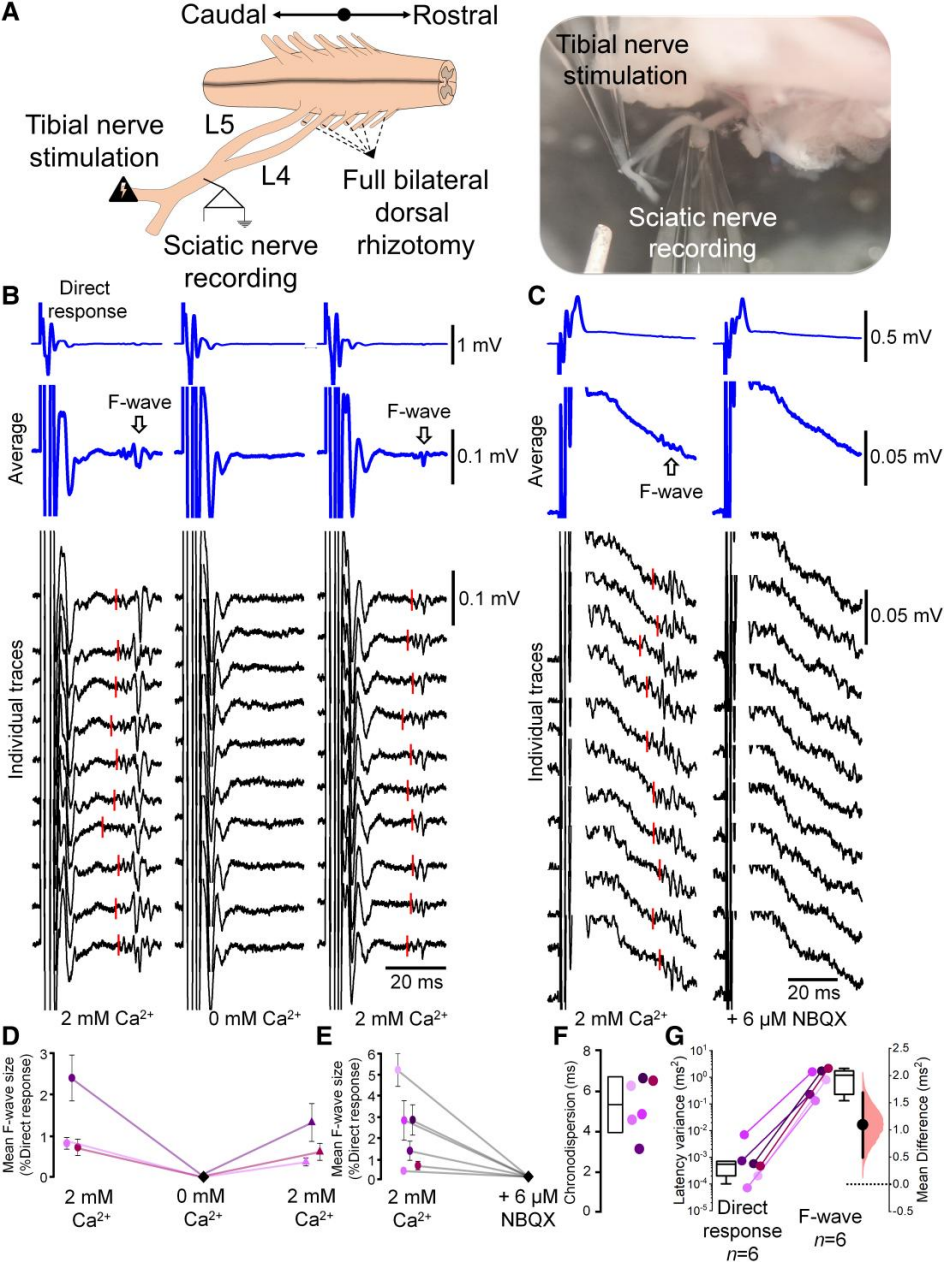
**Stimulation of the tibial nerve also elicits F-waves that depend on central glutamate release.** (**A**) Drawing (*left*) and photograph (*right*) of the isolated spinal cord–sciatic/tibial nerve neonatal mouse preparation, depicting stimulation (tibial nerve) and recording (sciatic nerve) sites. (**B** and **D**) Representative averaged (blue trace, without peak alignment) and individual (black) traces obtained from a 2-day-old mouse pup, showing the direct responses (*top row*). These traces are increased in amplification (*second row*) to show the F-wave responses (indicated by arrows). The F-wave recorded in the presence of 2 mM extracellular Ca^2+^ (*left*) was abolished in Ca^2+^-free artificial CSF (*middle*) and recovered following reperfusion of 2 mM Ca^2+^ solution (*right*). Red lines show the estimated onset of F-waves. (**C** and **E**) The AMPA receptor antagonist, NBQX (6 µM), abolished the F-wave (different preparations from **B**). Red lines show the estimated onset of F-waves. (**F**) F-wave chronodispersion at control Ca^2+^ levels (mean ± standard deviation). (**G**) The latency variance of the direct response and F-wave shows high variance in F-wave latencies (note log_10_ scale). *n* = number of *ex vivo* preparations (animals). *Right*: Effect size estimation plot.

In clinical studies, the F-wave is usually evoked by stimulating a single branch of the sciatic nerve. We therefore repeated our experiments stimulating only the tibial nerve and recording from the sciatic nerve using the same stimulation paradigm as above (Fig. 3A). Similar to the previous set of experiments, following tibial nerve stimulation, we detected an initial direct response, followed by a long latency event (37.1 ± 6.4 ms, CV: 0.42 ± 0.01 m/s for *n* = 2), smaller in size and with clear chronodispersion of 5.3 ± 1.3 ms (P1–3, *n* = 6; Fig. 3B and F) and high latency variability (Fig. 3G). This showed that stimulation of a single branch of the sciatic nerve, in a configuration similar to that used during clinical tests, is sufficient to evoke the *ex vivo* analogue of the F-wave.

To confirm the synaptic identity of the F-wave recorded at the sciatic nerve following tibial nerve stimulation, we repeated the experiments, blocking either pre-synaptic release or post-synaptic receptors. Blocking pre-synaptic release by removing Ca^2+^ did not affect the direct response (∼1% increase compared with control condition) but resulted in complete suppression of the F-wave, which was then restored upon reapplication of normal artificial CSF containing 2 mM of Ca^2+^ (Fig. 3B and D). Similarly, blocking AMPA receptors with NBQX (6 µM) completely abolished the F-wave without affecting the initial response (∼4% reduction compared with control condition; Fig. 3C and E). Altogether, these experiments showed that the clinically-relevant analogue, stimulation of a branch of the sciatic nerve (i.e. tibial nerve), is sufficient to generate the F-wave *ex vivo*, and that the F-wave is abolished by blocking either transmitter release or post-synaptic AMPA receptors. That is, the F-wave results from synaptic activity.

## Discussion

We used an *ex vivo* neonatal mouse spinal cord preparation with the sciatic nerve intact and showed that antidromic activation of motor axons from either the whole sciatic nerve or its posterior branch (tibial nerve) can evoke relatively long latency responses in the sciatic nerve. This response has the fundamental electrophysiological signatures of the F-wave.^2^ We demonstrated that this F-wave is abolished by blocking synaptic transmission by either removing extracellular Ca^2+^ or by blocking post-synaptic glutamate receptors. These observations indicate that the F-wave is synaptically generated by glutamatergic spinal microcircuits activated by synchronous motor neuron firing.

In clinical settings, low intensity stimulation of a mixed nerve generally evokes an initial H-reflex in the muscles. The gradual increase in the intensity initially results in a larger H-reflex and in activation of motor axons, such that a direct motor response (M-response) is generated. However, further increment in intensity leads to an increase in the M-response, while the H-reflex decreases in amplitude until it is completely abolished due to collision of the antidromic and orthodromic motor volley.^19^ After this stage, an F-wave appears, characterized by low amplitude, variable shape and high jitter. F-waves result from activation of motor axons, possibly those innervating fast twitch muscle fibres.^20,21^ Sensory afferent activation is not required, as the F-wave can still be obtained in deafferented patients or in animals with severed dorsal roots.^22^ However, despite being widely used, the physiology of the F-wave is not yet understood.

**Figure 4:**
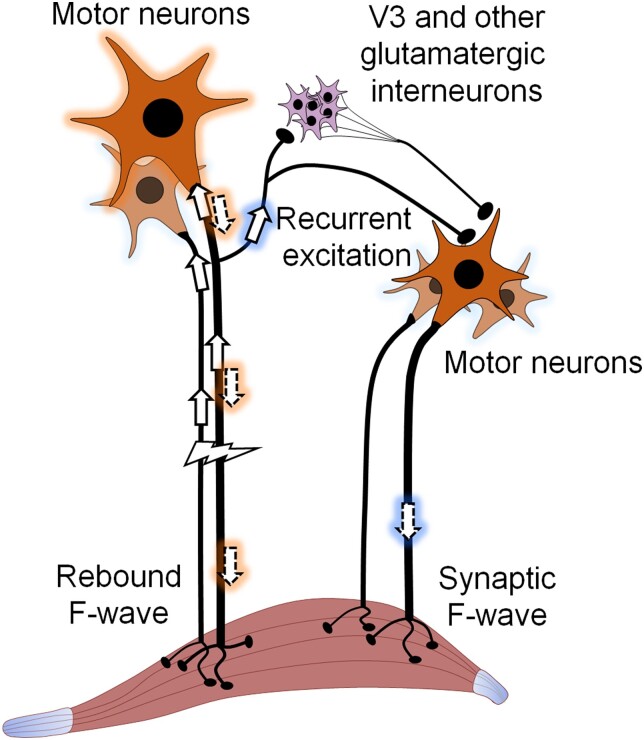
**Possible mechanisms behind the F-wave.** Classical explanation is that the stimulation of motor axons antidromically leads to the re-activation of some of the motor neurons, which then triggers a ‘rebound’ response (*left*). This response is recorded in the muscle as the F-wave. The mechanism proposed here is based on central synaptic glutamate release. The stimulation of motor axons leads to the generation of action potentials in some motor neurons through recurrent excitatory collaterals, activating either mono- or disynaptic excitatory microcircuits (V3 interneurons or other oligo-synaptic pathways), resulting in a ‘synaptic’ F-wave. The motor neurons generating the synaptic F-wave are also stimulated, but for representative purposes this is not shown on the figure.

The commonly accepted idea behind the F-wave is that stimulation of motor axons leads to re-excitation of the somatodendritic membrane that subsequently results in the stimulated motor neuron re-firing, giving rise to a ‘rebound’ F-wave (Fig. 4, left).^10,23-25^ The variable shape of the F-waves across trials was attributed to different motor neurons producing the rebound firing in different trials.^26^

Here we show that, at least in *ex vivo* preparations using nerve recordings (which should be no different than EMG recording due to the high safety factor of neuromuscular junctions), F-waves result from efferent-triggered central glutamate release. Antidromic invasion of motor axon collaterals can activate various circuits, including inhibitory Renshaw cell recurrent loops^27^ as well as multiple excitatory microcircuits. One of these excitatory loops is made by motor axon collaterals forming glutamatergic synaptic connections with other ipsilateral motor neurons.^12,13,28^ Recurrent motor axon collaterals also activate a ventrolateral population of V3 interneurons, which in turn form glutamatergic synapses with motor neurons.^17^ In addition, other circuits receiving motor collateral inputs such as ventral spinocerebellar tract neurons^15^ and currently unidentified locomotor circuit neurons,^16^ could also contribute to F-wave shape and duration variability by providing recurrent oligo-synaptic inputs to motor neurons. Of note, motor neuron to motor neuron connections are maintained beyond the neonatal stage and span beyond a single spinal segment.^12^ These connections are 10 times greater in magnitude in large post-synaptic motor neurons innervating fast twitch muscle fibres compared to small ones that innervate slow twitch fibres.^12,28^ As such, synaptically-generated F-waves, as shown here, would be expected to be predominant in large motor neurons—a suggestion compatible with the clinical observation that F-waves are generated primarily by larger motor units.^20,21^

In our *in vitro* conditions (Fig. 1), as well as in all the *in vivo* recordings we are aware of, somatic invasion of the antidromic spike prevents the generation of an early orthodromic spike. In fact, an SD spike will prevent a second axonal spike generated by intracellular current injection unless the motor neuron is hyperpolarized to a point where there is a significant IS-SD delay.^29^ If the motor neuron is relatively hyperpolarized at the time of arrival of an antidromic impulse (evoked by peripheral stimulation), the IS spike may not activate the SD membrane.^30^ In this case, the recurrent EPSP could be sufficient to generate an orthodromic spike that, if sufficiently delayed beyond the absolute refractory period of the axon,^29^ would then propagate towards the muscle (Fig. 4, right). With F-waves being produced via orthodromic (synaptic) activation, they will be particularly sensitive to membrane voltage, because the voltage would need to be hyperpolarized sufficiently to block the SD spike, and yet not so much that the EPSP does not reach threshold. In fact, it has been suggested that F-waves reflect the state of motor neuron inhibition,^6^ a condition that could lead to SD spike failure. In awake animals, motor neuron membrane potentials fluctuate, meaning that at the given moment of stimulation, a variable pool of motor neurons will participate in the F-wave. That is, fluctuations of membrane voltage could account for both the observed variability in amplitude and the chronodispersion, because the stochastic nature of these events would make it unlikely that the same motor neurons (with the same conduction velocities) would be recruited from trial to trial.

One limitation of our study is that we were restricted to studying neonatal animals. We note that recurrent excitatory connections are seen in mature preparations. Furthermore, although motor neuron excitability and membrane properties differ between neonatal, juvenile and adult mice,^31^ and synaptic inputs change throughout development, we found no evidence of backfiring in our *in vitro* recordings towards the end of the second postnatal week (Fig. 1), at a time when motor neuron properties reached an advanced stage of maturation.^31^ Similarly, backfiring has not been reported in adult motor neuron recordings following antidromic invasion.^11^

Could understanding the physiology of F-waves provide any further insight into the pathophysiology of disease? For example, in people with ALS, the F-wave has reduced persistence but increased amplitude, latency and chronodispersion. Each of these effects can be explained by ‘rebound’ action potentials as well as by orthodromic synaptic activation. But if synaptic activation is necessary for F-waves, perhaps the recurrent excitation of slow motor neurons as well as inhibitory inputs (to hyperpolarize motor neurons) both increase (supplementing a homeostatic response^32^) as the disease progresses. That is, the F-wave may be sensitive to changes in synaptic inputs to motor neurons across the course of the disease.

In summary, our results indicate that the F-wave is synaptically-mediated by recurrent excitation through motor axon collaterals. Our findings provide further evidence that the F-wave reflects not only the excitability of motor neurons but also their synaptic connectivity patterns. Both of these parameters may be affected in neurological disorders and may thus impact F-waves.

## Supplementary Material

awad342_Supplementary_Data

## Data Availability

Data used in this study are available within the article and its Supplementary material.
