# Supplementary material for: On the origin of F-wave: involvement of central synaptic mechanisms
Source: Brain. 2023 Oct 5;147(2):406–13. doi: 10.1093/brain/awad342 (PMC10834253; doi:10.1093/brain/awad342)
Supplement: awad342_Supplementary_Data [file awad342_supplementary_data.zip › brain-2023-01139-File006.pdf]

Supplementary Material

Materials and Methods

Dorsal horn ablated spinal cord preparations

We performed whole-cell recordings from labelled motor neurons in dorsal horn ablated preparations using the method we have previously reported.[1] Briefly, P2-6 days old mice were anesthetized using isoflurane and we injected ankle dorsiflexor tibialis anterior (TA) or plantar flexor gastrocnemius (GS) muscles with Cholera Toxin Subunit B (CTB) conjugated Alexa Fluor 488 or 555 (1 µL and 0.2% wt/vol), respectively, to retrogradely label motor neurons. After 2-7 days, mice were anesthetised through intraperitoneal injection of a ketamine/xylazine mixture (at doses of 100 mg/kg and 10 mg/kg, respectively), and decapitated. The spinal cord was then dissected in ice-cold artificial cerebrospinal fluid (aCSF) containing (in mM) 113 NaCl, 3 KCl, 25 $NaHCO_3$, 1 NaH_2PO_4, 2 $CaCl_2$, 2 $MgCl_2$, and 11 D-glucose and continuously bubbled with 95% O_2 and 5% CO_2. L3-L5 ventral roots-attached spinal cord was placed in agar block ventral side facing up, longitudinally cut using vibratome (HM 650 V, Microm, Thermo Fisher Scientific, UK) and incubated in a chamber with aCSF at 37°C for 30 mins, and kept at room temperature (~21ºC) during electrophysiological recordings.

Ex vivo spinal cord preparations with nerves attached

Animals were anesthetised through intraperitoneal injection of a ketamine/xylazine mixture (at doses of 100 mg/kg and 10 mg/kg, respectively), and decapitated. Mice were skinned and eviscerated with hindlimbs attached, keeping the spinal column intact. Following mid-thoracic spinal transection and vertebrectomy, the sciatic nerve, and triceps surae muscles were dissected in ice-cold aCSF. The spinal cord was gently lifted using a glass pipette to gain access to the dorsal side to perform full bilateral dorsal rhizotomies. Ventral roots were also cut, sparing L4 and L5. The common peroneal nerve and the tibial nerve branches were identified and dissected until the hip joint. The *ex vivo* spinal cord with sciatic nerve attached (Figs. 2A and 3A) was then immersed in aCSF which was continuously bubbled and kept at room temperature (~21ºC) before being used for electrophysiological recordings.

Electrophysiology

Patch clamp recordings were performed using an Axopatch 200B amplifier (Molecular Devices, Sunnyvale), low pass filtered at 5 kHz, digitised at 50 kHz with a Digidata 1440A A/D board (Molecular Devices), and acquired with Clampex 10 software (Molecular Devices). *Ex vivo* nerve recordings were performed using an NPI Ext-02F amplifier (NPI electronic GmbH) and signals were bandpass filtered at 1Hz – 10 kHz. Borosilicate glass (GC150F, Harvard Apparatus) electrodes for recording and stimulation were pulled with a Flaming-Brown puller (P1000, Sutter Instruments), cut to ~1.5x the root or nerve diameter for stimulation and nerve recordings, attached to a head stage (Narishige), filled with regular aCSF, then attached to roots by suction. Electrical current was delivered using a DS3 isolated current stimulator (Digitimer). Patch recordings were performed using glass pipettes (∼1–4 MΩ resistance) filled with intracellular solution containing (in mM): 125 K-gluconate, 6 KCl, 10 HEPES, 0.1 EGTA, 2 Mg-ATP, pH 7.3 with KOH, and osmolarity of 290–310 mosmol/kgH_2O. Membrane potentials were reported without correcting the junction potential for the intracellular solution (∼15 mV).

In vitro spinal cords were placed in a chamber continuously perfused with aCSF at room temperature and imaged using Eclipse E600FN Nikon microscope (Nikon, Japan). Infrared differential interference contrast images were captured with a digital camera (Nikon, DS-Qi1Mc), while fluorescence was detected through a laser scanning confocal unit (D-Eclipse C1, Nikon) equipped with two diode laser lines (λ = 488 and 561 nm). The stimulus intensity was defined by the threshold intensity, which is the minimum intensity that generated excitatory postsynaptic potentials (EPSP) reliably in motor neurons. Both L4 and L5 ventral roots were stimulated with 2-3x threshold intensity. Labelled motor neurons (n=11 TA, n=8 LG) were patched (Fig. 1A-B) and stimulus-evoked responses were recorded in the current-clamp configuration. The occurrence of antidromic and orthodromic spikes at different resting potentials were tested in 25 unlabelled motor neurons (Fig. 1C).

The spinal cord–sciatic nerve *ex vivo* preparation was placed in a chamber with aCSF continuously perfused at 5 mL/min. Either the tibial or whole sciatic nerve was stimulated, preferentially from a distal location along the isolated nerve, with the nerve recordings being performed with a suction electrode positioned on a proximal section of the sciatic nerve (see Figs. 2A and 3A). The nerve was stimulated every 15 seconds with 0.2-0.5 ms pulse width. Stimulus intensity was set at around 10% above the intensity that generated the maximum early direct response measured from the sciatic nerve. We refer to this early response as the “direct response,” recognising that it is not equivalent to the direct response recorded clinically from

the muscle as sensory axons will also be stimulated; we used this direct response as a reference to test the stimulation efficiency throughout the experiment. That is, we constantly monitored this direct response and the F-wave from the sciatic nerve (or tibial branch), and the experiments were terminated if the reduction in the direct response exceeded 20%. Throughout the text, we have reported the latency and chronodispersion (the difference between the maximum and minimum F-wave latency) of the F-wave response as the mean ± standard deviation usually obtained from ~10 or more individual traces. The average size of F-waves was calculated by measuring peak-to-peak amplitude of F-waves in each sweep and then averaging these values. The conduction velocity (CV) was calculated as follows; the distance from the stimulation site to the cord and back to the recording (in mm) site was divided by the latency (ms) of the F-wave. Manipulation of the synaptic excitability was done by lowering extracellular Ca^{2+} to 0 mM or exogenous application of the α-amino-3-hydroxy-5-methyl-4-isoxazolepropionic acid (AMPA) receptor antagonist - 3-Dioxo-6-nitro-1,2,3,4-tetrahydrobenzo[f]quinoxaline-7-sulfonamide disodium salt (NBQX) - at a concentration of 6 µM that is effective to block the AMPA receptor mediated currents. Graphs showing the mean size of the F-wave and chronodispersion were plotted using OriginPro 2021 (OriginLab Corporation, Northampton, MA), and effect size estimation plots[2] were plotted with MATLAB R2022a (Mathworks, Natick, MA).

Data availability

Data used in this study are available within the article and its Supplementary material.

References

1. Özyurt MG, Ojeda-Alonso J, Beato M, Nascimento F. In vitro longitudinal lumbar spinal cord preparations to study sensory and recurrent motor microcircuits of juvenile mice. J Neurophysiol. Sep 1 2022;128(3):711-726. doi:10.1152/jn.00184.2022

2. Ho J, Tumkaya T, Aryal S, Choi H, Claridge-Chang A. Moving beyond P values: data analysis with estimation graphics. Nat Methods. Jul 2019;16(7):565-566. doi:10.1038/s41592-019-0470-3
